# Evaluating the Impacts of Health, Social Network and Capital on Craft Efficiency and Productivity: A Case Study of Construction Workers in China

**DOI:** 10.3390/ijerph15020345

**Published:** 2018-02-15

**Authors:** Jingfeng Yuan, Wen Yi, Mengyi Miao, Lei Zhang

**Affiliations:** 1 Department of Construction and Real Estate, Southeast University, Nanjing 210096, China; mmyMiaow@163.com (M.M.); zl0930@yeah.net (L.Z.); 2 School of Engineering and Advanced Technology, Massey University, Auckland 0632, New Zealand; W.Yi1@massey.ac.nz

**Keywords:** structural equation model, physical and mental health, social network, social capital, craft efficiency, craft productivity

## Abstract

The construction industry has been recognized, for many years, as among those having a high likelihood of accidents, injuries and occupational illnesses. Such risks of construction workers can lead to low productivity and social problems. As a result, construction workers’ well-being should be highly addressed to improve construction workers’ efficiency and productivity. Meanwhile, the social support from a social network and capital (SNC) of construction workers has been considered as an effective approach to promote construction workers’ physical and mental health (P&M health), as well as their work efficiency and productivity. Based on a comprehensive literature review, a conceptual model, which aims to improve construction workers’ efficiency and productivity from the perspective of health and SNC, was proposed. A questionnaire survey was conducted to investigate the construction workers’ health, SNC and work efficiency and productivity in Nanjing, China. A structural equation model (SEM) was employed to test the three hypothetical relationships among construction workers’ P&M health, SNC and work efficiency and productivity. The results indicated that the direct impacts from construction workers’ P&M health on work efficiency and productivity were more significant than that from the SNC. In addition, the construction workers’ social capital and the network can indirectly influence the work efficiency and productivity by affecting the construction workers’ P&M health. Therefore, strategies for enhancing construction workers’ efficiency and productivity were proposed. Furthermore, many useable suggestions can be drawn from the research findings from the perspective of a government. The identified indicators and relationships would contribute to the construction work efficiency and productivity assessment and health management from the perspective of the construction workers.

## 1. Introduction

The construction industry features high-level risks on the safety and health of the working population. Significant attention should be given to the safety and health of construction labourers by the research community and governments. The goal of the health, safety and environment (HSE) management is to make work adapt to workers and workers adapt to their work. Meanwhile, promoting and maintaining the highest happiness of all the workers in physical, mental and social activities should be further realized [1]. Therefore, workers should be prevented from losing their health because of unsafe working conditions and be protected from harmful factors from their working environment [2,3]. In addition, work efficiency and productivity would be improved if the workers can adapt to the work environment physically and mentally [4]. In recent years, many kinds of improvements on workers’ health and their psychosocial work-environment have been launched to increase productivity and profit in different industries, including the manufacturing industry, IT (information technologies) industry and construction industry [5]. Although many measures (e.g., reducing operation costs, reducing employee turnover, lowering healthcare coverage, etc.) can be adopted to improve their work efficiency and productivity, a workforce with high physical and mental health (P&M health) should directly and positively affect the efficiency and productivity [6].

As a tough, heavy and manual industry, the construction industry is one of the most dangerous sectors around the world [7]. Physical injuries and illnesses always cause the reduction of craft productivity, work enthusiasm and an increased absenteeism rate. In this case, the work efficiency and productivity improvements stem from the expectation that a healthier workforce requires less input, produces more output of better quality and performs better [6]. On the other hand, the construction industry also could have a negative effect on the psychological well-being of workers due to a plethora of occupational demands in construction projects, which could further have an adverse influence on individual and organizational performance [8]. Therefore, construction workers’ efficiency and productivity can be improved by focusing on the physical health and mental health [9,10].

Furthermore, construction work efficiency and productivity improvements are correlated with the social support of construction workers [8]. It was indicated that social barriers to communication can affect economy-wide productivity and factor accumulation [11]. The communication and relationship between different individuals or tasks can be expressed by a social network and capital (SNC). A social network is a social structure made up of a set of social actors (such as construction workers), sets of dyadic ties and other social interactions between actors. Actually, humans are a type of social creature due to living in a community. Meanwhile, individual social networks could influence their behaviour [12]. Thus, norms and habits would spread through the social networks. Moreover, the social network represents the individual’s social capital, which is the collective value of all the social networks. This value arises because a network allows accomplishing an important mission and improving work efficiency and productivity [13]. Therefore, a perfect social network can positively influence the work efficiency and productivity.

With the extensive workforce that the construction industry employs, health- and safety-related issues in the construction industry have become important, since the industry is still the one with the highest fatality and accident rates [14]. Therefore, health and safety management in the construction industry should be highly enhanced, thereby helping construction organizations achieve their H&S objectives. However, while many studies have been conducted to improve the safety performance of construction projects, few studies have focused on the health management aspect. The relationship between health management and work outcomes has not been well examined [15,16]. Furthermore, the influence of an SNC on work performance also should be identified in order to help construction organizations reduce work pressure, improve the social capital and improve work efficiency and productivity [17,18,19].

Accordingly, in order to analyse the relationships among construction workers’ efficiency and productivity, workers’ health and their SNC, two ways are considered within a construction project to improve the construction workers’ efficiency and productivity including the improvement of P&M health and the enhancement of SNC for construction workers. As a result, the study aims to investigate the different ways and related measures to improve construction workers’ efficiency and productivity through a literature review and examination of the construction workers’ perception of the workers’ efficiency and productivity from the perspective of health and SNC in China. A conceptual model was proposed based on previous studies for explaining the theoretical relationships between those dimensions (ways), and an SEM model was developed by using questionnaire survey data to test the proposed methods for improving workers’ efficiency and the productivity of a construction project.

## 2. Background

### 2.1. Literature Review

#### 2.1.1. Workers’ Efficiency and Productivity in Construction

Compared to other industrial sectors, construction is considered less progressive [20]. The need for improvement in terms of efficiency and effectiveness (e.g., time, costs and quality) of work is often discussed, for instance in programmes of continual improvement or in generative learning [21,22]. Hence, the most challenging issue in the construction industry in the last two decades is how to improve the production efficiency and productivity.

Many prior studies have been done; however, a deeper understanding is still needed to improve the efficiency and productivity by studying the different ways that could influence the workers’ psychology and behaviour in the construction industry [14,23,24,25]. Actually, the performance of labour is usually linked to the performance of time, cost, work pressure, safety measures and quality, which would be affected by many factors including management, environment, technologies and their own situation [23,26]. For the management-related issues, prior studies focused on project management (e.g., delay, absenteeism, payment, unreasonable project goals and milestones, decision-making, etc.), supervisor direction and safety management [27,28,29,30]. For the environment-related issues, prior studies paid much more attention to the work environment including the workplace, work team and foreman [31,32,33,34,35]. For the technology-related issues, prior studies aimed at advanced technologies or technical innovations, tools, material, engineering drawing and equipment [36,37,38]. For the workers’ own situation, prior studies were related to human problems, including communication problems, availability of health management, lack of pride in their work, lack of incentive to attend work and training and lack of incentive for good performance [39,40,41]. Productivity is positively correlated with safety [42].

#### 2.1.2. Health Management in Construction

The safety, health and environmental issues of construction projects have become increasingly prominent, and protecting the safety of employees and their health needs to address the main problem effectively [43]. The HSE management system is usually used to reduce the accident severity rate. However, the system associated with HSE and HSE risk management established only 41.8% and 18.4% respectively, according to the data gathered from comprehensive accident investigation reports [44]. There is still a gap between the application of HSE knowledge and the learning outcomes for the civil engineering program [45].What is more, the researches analysing the accidents and their factors and solutions are always focusing on safety, more than the environment, and, at least, workers’ health. For example, the system associated with HSE and HSE risk management only identified workers’ age, job experience, activity type, periodic training, duration and content of training [44].

When it comes to the safety management in construction, prevention through design (PtD) is an emerging concept for reducing safety hazards and worker injuries [46]. By designing out the hazards and risks, occupational illnesses and injuries can be prevented and controlled during work [47]. However, the existence of economic, legal and contractual obstacles to practice PtD cannot be ignored by the participants in construction projects, which argues for more PtD education and training in safety management [48]. The United Nations issued Transforming our World: The 2030 Agenda for Sustainable Development in September 2015, which included 17 targets being divided into five categories including people, Earth, prosperity, peace and partners [49]. The third one is to ensure a healthy lifestyle and promote the well-being of all people of all ages. When it comes to workers’ health, occupational health should not be ignored. The definitions of occupational health vary from person to person. The most authoritative one is that: Occupational health should aim to promote and keep the physiological status, psychological status and social status of workers in different industries at its best [50,51,52]. Prior studies always focus on the ways of inducing the occurrence of health problems in construction [53], examining and promoting construction workers’ P&M health [54,55].

The health management in construction includes physical health management and mental health management. For physical health management, many prior studies focused on discussing the relationship between physical health and safety, including integrating safety and health performance into construction management [56,57,58], and analysing critical factors related to physical health on construction workplace safety management [7,30,59]. Therefore, many methods preventing construction worker injury incidents through the management of personal physical stress and organizational stressors are proposed, including reducing daily working hour and years, as well as improving rest time, sleeping time and the frequency of physical examination [60,61,62,63,64]. For mental health management, construction workers’ occupation and the surrounding context affecting their mental health could be grouped into four key themes: the importance of relationships, the impact of lifestyle, work characteristics, and mental health attitudes [53,65,66]. Meanwhile, the establishment of worksite health improvement programs can enhance the overall health of workers by developing or strengthening existing organizational health promotion, worker safety, self-protection, emotional control and disease prevention [67]. Moreover, workers’ health may be linked to a way of maintaining high levels of efficiency and productivity [68].

#### 2.1.3. Social Network and Capital for Individuals

Social network analysis has aroused increasing attention in construction project management research [69]. Social capital generally refers to the value of tangible and intangible resources, as well as the relationships among these resources, which generally can be viewed as a form of capital that produces public goods for a common good. Social capital has been used to explain the improved performance of different groups. The social capital theory has been extensively applied in the field of social science, organization management, human resource management, education management and knowledge management [70,71]. In construction social capital, the social capital theory is used to facilitate the construction innovation, knowledge sharing and performance improvement [72,73,74]. Social capital can be viewed as a primordial concept, which would affect project participants’ interactions and construction project performance [75,76].

Furthermore, social capital is a form of economic and cultural capital in which social networks are central. The social network can be expressed by multidimensional relationships between construction workers and construction enterprises (e.g., health training and different health protection measures by companies), friends or family (extensive social relationship with others) and society (government check on the construction site) [77,78]. These relationships represent the extensive social supports from different dimensions for construction workers. For example, safety-related communications are vital to include every individual in a construction crew to ensure strong safety performance [79,80]. It is better that the communication is involved in pre-construction decision-making [81]. Kulkarni also indicated that the workforce in the construction sector was the most vulnerable because employment was permanently temporary, the employer and employee relationship was very fragile and most of the time short-lived and the work had inherent risk to life and limb due to lack of safety, health and welfare facilities, coupled with uncertain working hours [82]. Therefore, many prior studies proposed that psychosocial factors should be considered in construction management to improve the project performance, including safety, health and productivity [23,76,83,84].

#### 2.1.4. Knowledge Gap

According to the above-mentioned literature review, prior studies mainly analysed the work productivity and efficiency, health management, social capital and social network in construction separately. Although many prior studies discussed factors influencing the work productivity and efficiency, few prior studies analysed relationships among work productivity and efficiency, health management and social supports [23]. Additionally, the human problems leading to the decrease of work productivity and efficiency should be further studied. Meanwhile, prior studies have identified the important factors affecting construction workers’ health. However, the influences of workers’ health on work efficiency and productivity have not been systematically reviewed. Although workers’ health does not constitute a distinct unit within the network of production, it may still influence and contribute to overall firm productivity [6]. Moreover, few prior studies focused on the contribution of social capital and network to work efficiency and productivity, as well as workers’ health has not been extensively reviewed. Therefore, the paper will focus on the relationships among work productivity and efficiency, health management, social capital and social network.

### 2.2. Conceptual Model and Hypothetical Relationships

The WHO rose in 1995 that the goal of occupational health should be: To promote and maintain the highest happiness of all the workers in physical, mental, and social activities. In addition, relevant studies indicate that good health is associated with higher overall performance [85]. Moreover, key factors for inefficient practices were identified to be communication skills and a narrow decision space that constrains the authority of district health managers and their ability to influence decision-making, which shows the significance of social network.

**Figure 1:**
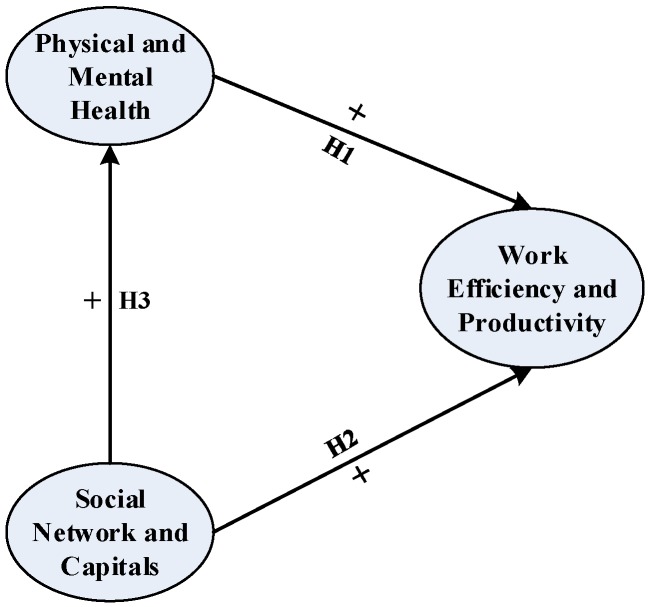
Proposed conceptual model.

As a result, physical health, psychological health and social relation network are defined as latent variables. The limitation of recognition due to the lack of observable variables could be avoided only through related estimates (namely add a new observable variable). Therefore, a new latent variable called work efficiency and productivity is introduced to quantitatively describe the influence on workers’ performance. The concrete relationships between the latent variables are shown in Figure 1.

**Hypothesis 1.**  *P&M health has a positive influence on work efficiency and productivity*.

Construction workers are exposed to a wide variety of health hazards at work. Both P&M hazards are exist. Therefore, construction workers in China suffer a disproportionate share of work-related injuries and illnesses. Obviously, physical injuries and illnesses could heavily reduce the work efficiency and productivity of construction workers due to accidents, lack of self-protection awareness, age, etc. [86,87]. Many occupational health problems are related to different kinds of workers (e.g., pneumoconiosis of the tunnel builder and the welder; low-back pain of the bricklayer; kidney ailments of the painter and the roofer from exposure to solvents; asbestosis of the building demolition worker; and heat stress of the hazardous waste clean-up worker) [57,88].

Meanwhile, the adverse effects of work could stress the individual and reduce work performance. For construction workers, work-related stress can contribute to P&M disorders [30,89]. Physical illnesses may include high systolic blood pressure, high cholesterol and stomach ulcers. Poor mental health can include low self-esteem, job dissatisfaction and job-related tension, and prolonged work-related stress can result in anxiety and depression. According to previous research, the above-mentioned P&M disorders could further reduce the work efficiency and productivity of construction workers [90,91]. Therefore, inadequate health has a substantial negative effect on labour efficiency and productivity. The adverse effects on individual well-being can often have a detrimental impact on an organization including increased staff turnover and absenteeism and reduced efficiency and productivity.

**Hypothesis 2.**  *The SNC has a positive influence on work efficiency and productivity*.

In China, most of the construction workers are from rural areas and permanently temporary, frequently changing employers. Usually, most construction projects require living in work camps that are away from construction workers’ home and family [92]. There is no recreational facility, lack of access to education for children, poor sanitary facilities and a lack of safe drinking water at the construction site [93]. The work stress could increase in the construction process because of the heavy workload, possible violence in the workplace and limited social supports [94]. The related addictions to alcohol, tobacco and smoking would contribute to illness and suffering [95,96]. These problems related to the social relationships of construction workers would negatively influence the work performance.

In addition, it was demonstrated that a construction project is a network-based organization composed of different participants with different expertise at different times [97]. The social capital of the group can be delivered by the relationships among the participants [98,99]. Moreover, a project social network is generated by project participants and their relationships. Therefore, the social capital of construction workers is supported by the social network of construction workers and other project participants. The contribution of the social network and capital of construction workers to work efficiency and productivity can be identified by the interdependence arising from the interconnections between construction workers and other workers, construction enterprises and their families or friends [41,99]. Actually, construction workers need to rely on others to complete tasks, which can help construction workers promote the social network and capital by increasing the opportunity of participants’ interactions to improve the work performance [100]. Moreover, these interactions of the project organization provide the opportunity for sustained socialization, which is essential for the creation and maintenance of networks and social relationships [100]. Meanwhile, Rizova indicated that group dynamics could be used to describe the construction workers’ interactions and the patterns of social relations established by the construction workers [101]. Hence, the social network and capital engendered among the construction workers would affect the group performance, and ultimately the project performance.

**Hypothesis 3.**  *The SNC has the positive influence on P&M health*.

Weiss found that workload was a predictor of workers’ well-being, as it was positively related to psychological and physiological strains [102]. The relationship among psychological stressors at work and adverse health outcomes and the need to develop coping strategies are influenced by the effects of social support among construction workers [103]. The social supports are strongly related to the social network and capital and can be measured as the perception that one has assistance available, the actually received assistance or the degree to which a person is integrated into a social network [15].

Social relationships may influence health outcomes by influencing the practice of health-related behaviours, including preventive and lifestyle behaviours, treatment adherence and illness-management behaviours. It was indicated that social problems could greatly influence the mental health of construction workers [8,104]. In order to cope with social problems, social network and capital should be enhanced to improve the social support for construction workers. The social supports can be further defined as the existence or availability of people, who can be relied on by construction workers and who are a source of self-validation [105]. The theory of Ostermann also suggested that social supports would have an effect on both P&M health and that consideration should be given to indicators of good mental health to assist in the identification of positive practices [106].

## 3. Research Methodology

### 3.1. Research Design

**Figure 2:**
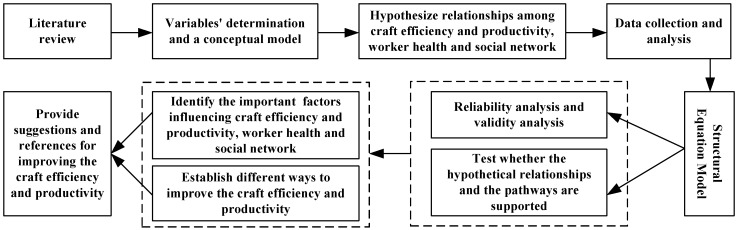
The research methodology.

In order to measure the extent to which construction workers’ health and SNC influence work efficiency and productivity, the hypotheses raised were mapped into the causal relation model based on the literature review and the characteristics of workers’ health, SNC and work efficiency and productivity. In addition, the relevant indicators were identified. A questionnaire survey using a stratified random sampling method was then conducted to collect the data from construction workers to find the internal relationships among workers’ health, social network and work efficiency and productivity. The statistical analysis, Cronbach’s alphas, was performed using the SPSS 19.0 software (IBM, Armonk, NY, USA) package to validate the consistency of data. Moreover, the structural equation model was introduced to explore the concrete degree of influence that health status and social supports have on work efficiency and productivity. An SEM model can be used to test whether the conceptual model and the hypothetical relationships were supported based on survey data through AMOS 20 software (IBM, Armonk, NY, USA). The organization of the methodology adopted in the research is shown in Figure 2.

### 3.2. Initial Indicators Measuring Health, Social Capital and Work Efficiency and Productivity

Overall, P&M health, social relation network and work efficiency and productivity are defined as latent variables. After determining the latent variables, observed indexes should also be found to lay the foundation for modelling.

#### 3.2.1. Indicators Measuring Health

Construction workers are employed and asked to finish daily work and usually have to do many work tasks. Therefore, they do not have enough time to entertain themselves and relax. If they cannot adjust their mental condition well, this might easily produce emotional anxiousness and may increase safety risks. Combined with the prior literature review, the factors influencing construction workers’ P&M health can be described as follows. Increased self-protection consciousness (*a*1) is mentioned by Cohen and other experts as a possible way to job health and safety matters accompanied by close interactions with construction workers [107]. It was indicated that control over daily working hours (*a*2) might protect health and help workers successfully combine a full-time job with the demands of work [108]. Meanwhile, an appropriate work-rest schedule or time (*a*3) should be recognized as an efficient way of providing better ergonomic environment, improving labour health and productivity [35,109]. Relatively high frequency physical examination (*a*4) can provide timely health assessment for construction workers to help workers know and improve their health status [110]. Moreover, it was mentioned that construction workers’ performance in the following day would be certainly affected when workers’ sleeping time (*a*5) is less than 6 h per day [111]. Working years can represent the experiences and ages of construction workers, which could influence health. At the same time, different working years (*a*6) could bring different job stresses with statistically differences according to Mok and other experts [112], which would lead to different impacts on the workers’ mental health. Furthermore, Patrick demonstrated that negative emotions such as fear and anxiety could lead to abnormal risk-taking behaviours [113]. The occurrence of anxious and upset mood (*a*7) would guide human responses to dangerous situations [114], which means more frequent anxiety and fear would lead to more negative impacts on the workers’ mental health.

#### 3.2.2. Indicators Measuring SNC

Social network and capital should reflect the social supports and may have an influence on workers’ safety including all the social interactions. In terms of construction workers, their social network mainly includes interaction with the government, companies and individuals, such as regular checks by a government department, protection appliance provided by companies, specific occupational health lectures and their own social status. Therefore, the factors influencing social relation network and capital can be determined in detail. For the relationship with the government, the supervision of the government in China was identified as a key factor influencing safety and health management in construction [115]. Therefore, the frequency of checks by the government (*a*8) can reflect the degree that can facilitate the health management at the construction site and ensure the implementation of health protection and training. For the relationship with individuals, social relationships with others (*a*9) can represent the social status of construction workers, especially effective safety communication between all parties in a construction project, which could benefit the safety performance [116]. Problematical social relationships of construction workers were considered as important factors leading to communication barriers [117]. For the relationship with companies, active and passive health protection measures provided by companies (*a*10) and specific occupational health training (*a*11) can make construction workers understand the level of health risks in different situations and prevent the workers from health risks [84,118,119]. The health protection measures could include protection equipment, health insurance, health subsidies, vacations and physical examinations. Health training can enhance the needs of construction workers, which may have a positive effect on workers’ attitudes, work practices and self-reported injury rates [119].

#### 3.2.3. Indicators Measuring Work Efficiency and Productivity

The latent variable called work efficiency and productivity is introduced to describe the influence on workers’ performance quantitatively. Traditionally, time, quality and cost are three key performance indicators (KPIs) in project management [120]. Therefore, observed variables corresponding to work efficiency and productivity are the quality of finished work and work progress. On the other hand, Liden and other experts indicate a positive relationship between work attitude and job performance [121].

Based on the above-mentioned conceptual model and prior research, three latent variables and fourteen observed variables about construction workers were analysed through the literature review. They are described as follows in Table 1.

#### 3.2.4. Data Collection

The questionnaire was designed to survey construction workers’ perspectives on the aforementioned indicators. The questionnaire covered two parts. The first part was about the background information of the respondents, including gender, age and related experience at the construction site. The second part consisted of fourteen items using the Likert five-point scale measurement for the convenience of statistical analysis and SEM.

To ensure the consistency and accuracy of the questionnaires, the author conducted a pre-survey based on the questionnaire draft, which is a small sample investigation to revise, improve and validate the questionnaire. Formal random sampling was conducted for five construction projects in Nanjing, China. All of them are typical construction projects, where almost all the construction workers started working after finishing secondary school education. Thus, they do not share any differences except gender. The participants were chosen from different types of work and invited to make their own judgments on those items according to reality. Fourteen questions are shown in Table 2. The answers to the questions can be transferred to five scale intervals. A Likert scale is the most widely-used approach to scaling responses in survey research with a psychometric scale commonly involved in research that employs questionnaires [122,123].

In this survey, 150 questionnaires were sent out, and 133 valid samples were returned. The missing value was addressed by the listwise method, that is once there is a missing value in a record, then it is deleted from the record, which is commonly used in most statistical analysis software to improve data quality [121,123]. Finally, 118 records were left, and the effective rate was 78.7%. The respondents were workers for different construction projects. In the total of 118 respondents, only 12 respondents were female. The average age of all respondents was 40.27. Twenty respondents were 20–30 years; 44 respondents were 30–40 years; 42 respondents were 40–50 years; 10 respondents were 50–60; and 2 respondents were over 60 years. For the working experiences of respondents, 10 respondents have worked in construction less than 3 years; 24 respondents have worked 3–5 years; 54 respondents have worked 5–10 years; 12 respondents have worked 10–15 years; and 18 respondents have worked over 15 years. All the respondents’ educational levels are below primary school, and the socio-economic status is similar. Furthermore, an important hypothesis is that the opinions of respondents can be viewed as a whole though they may have different genders, ages and working experiences. Cronbach’s alpha should be used to support hypotheses.

## 4. Data Analysis

### 4.1. Data Analysis

#### 4.1.1. Reliability Analysis

Statistical tests were conducted to ensure that the sample could be treated as a whole and used for further analysis by SPSS 19.0. Reliability analysis was performed on the 116 valid questionnaires, and the result indicated high reliability (Cronbach’s alphas = 0.910, sig. = 0.000). It was noted that the threshold value of Cronbach’s alpha is 0.70, which is often acknowledged [124]. Secondly, reliability analysis can reflect the consistency and stability of results through Cronbach’s alpha, which ranges from 0–1. The closer the value approaches one, the higher the internal consistency of the data is. As shown in Table 3, Cronbach’s alpha if the item was deleted for working years (*a*6) is more than 0.910. This indicates that the reliability coefficient will increase if the item is deleted, which means the item has a low correlation with other factors. When the factor working years is deleted, the reliability coefficient of remaining 13 variables is 0.919. The obvious increase in the reliability coefficient shows that the removal of working years is rational.

#### 4.1.2. Validity Test

The validity test mainly relies on Kaiser–Meyer–Olkin (KMO) inspection and the Bartlett test of sphericity of valid data. It is generally believed that structural equation model can be carried out when the value of KMO is more than 0.5 and the Bartlett coefficient is below 0.05. In this survey, the value of KMO is 0.854, and the Bartlett coefficient is 0.000. Therefore, the overall validity of the questionnaire is high, and the confirmatory analysis of factors can be continued.

#### 4.1.3. Structural Equation Model

In this study, different advanced methods, such as SEM, have been applied to date in order to test relevant factors [125,126]. SEM is an estimating method that can handle a large number of exogenous and endogenous factors, as well as non-observed (latent) variables [127]. SEM, also called the simultaneous equation model, is a multivariate (multi-equation) regression model. These structural equations are meant to represent causal relationships among variables in the model [128]. SEM is frequently used in construction HSE management. Because safety performance in construction projects is attributed to many determinants (factors) in an HSE system, various directly- or indirectly-related determinants and their effects on HSE performance of construction projects should be examined by using SEM [129].

The structural equation model can handle multiple dependent variables at the same time and allows measuring error to exist in both dependent variables and independent variables. The SEM requires a theoretical model consisting of the measurement and structural models. The measurement model gives the relationships between the latent and observed variables, while the structural model shows the relationships among latent variables. The research model was analysed by using data from the above-mentioned survey, and AMOS 20 can be adopted to test the hypothesized relationships with satisfactory accuracy. Moreover, the SEM results should be assessed by the overall model fit. The index for the assessment includes χ^2^/degree of freedom (D*f*), the comparative fit index (CFI), the normal fit index (NFI) and the root mean square error of approximation (RMSEA). The recommended level of goodness-fit (GOF) measures are shown in Table 4 according to Holbert and Stephenson, Jashapara and Cognition [130,131]. Meanwhile, the questionnaire in this study involving 116 samples can meet the requirements of minimum sample size for conducting an SEM analysis in research. As presented by Sideridis and other experts, a sample size of 50–70 would be enough for SEM [132].

## 5. Results

**Figure 3:**
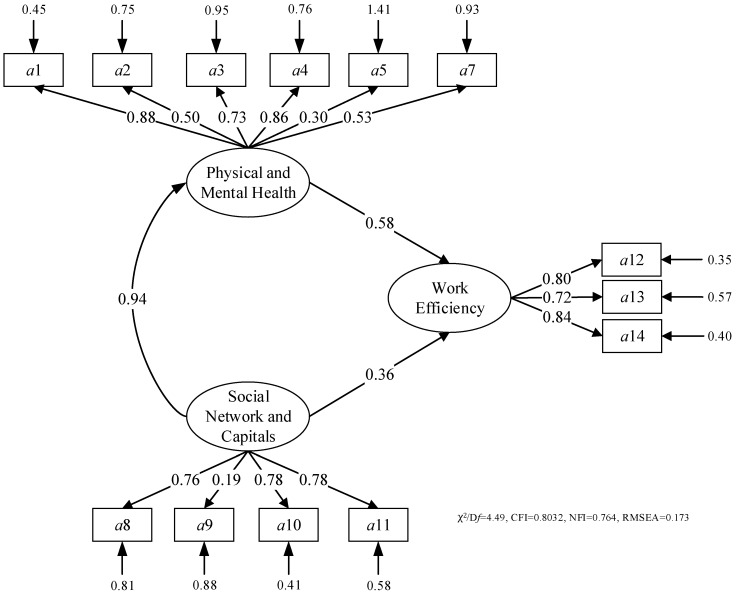
Pathway coefficients for the initial model for structural equation model (SEM).

The initial model showing the interrelationships between the latent variables is shown in Figure 3. Each of the latent variables and their corresponding factors are connected by one-headed arrows to indicate the direction of hypothesized influence. SEM is used to test the hypothetical relationships among latent variables in the initial model. In Figure 3, path diagrams are introduced to demonstrate the relationships between latent and measure variables by ovals and rectangles. Meanwhile, arrows can be used to connect the variables and represent the causal flow of relations. The regression relationships can be analysed by the one-headed arrows, where the direction of the arrow implies the direction of influence.

SEM is the most commonly used to test whether the survey data fit a hypothesized measurement model, which is based on theory and/or prior analytic research. Therefore, an SEM is conducted to test whether the proposed model fits the empirical data using AMOS, importing questionnaire data from SPSS. The conducted SEM may not be acceptable with insignificant indicators or other reasons, so it should be modified and improved. Thus, the adequacy of the initial SEM model should be determined, and the model’s fit to the data must be evaluated by the recommended level of goodness-of-fit (GOF) measures in Table 4 [133].

**Figure 4:**
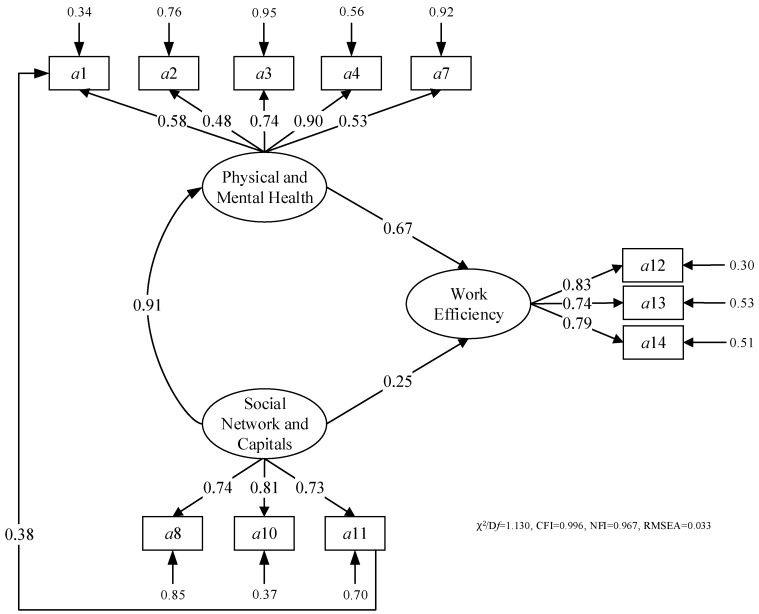
Pathway coefficients for the improved model for SEM.

The variables and the errors among the variables are presented in Figure 3 (initial model) and Figure 4 (improved model). The arrows and pathway coefficients (factor loadings) indicate the causal effect statistically and in terms of the relationship of variables and their corresponding factors reflecting the influences of P&M health, as well as social capital and network on the construction workers’ efficiency and productivity. The measurement and structural components are also shown in Figure 3 and Figure 4, demonstrating that the model directly reflects the relationships’ variables and their corresponding indicators.

SEM was performed to test the initial model, producing a parameter estimation and GOF of the initial model as shown in Figure 3. The estimates of pathway coefficients are presented in Figure 3. According to the estimation, the initial model, which includes all assumed factors and relationships (the factor *a*6 in P&M health is excluded according to reliability analysis), does not show a relatively good model fit (χ2/D*f* = 4.49, CFI = 0.803, NFI = 0.764, RMSEA = 0.173).

The results of the model evaluation are shown in Table 5. The model evaluation is to figure out whether the parameters estimated in the model have statistical significance, which is evaluated by the critical ratio (CR) value. CR is equivalent to the value of the *t*-test. While the absolute value of CR should be more than 2.58, the parameters are estimated at the 0.01 significance level, with *p* shown with “***”.

According to Figure 3, the factor *a*5 should be removed from the initial model as its error is larger than one. Prior studies indicate that sleeping time could influence the health of construction workers and further influence the work efficiency and productivity. However, at in the construction sites in China usually are from the countryside and living at the construction site, so they are required to work and sleep at the same time. Their sleeping time can be ensured to be more than 6 h, which would not significantly influence the construction workers’ performance the following day [111]. In this case, the impacts of sleeping time could be ignored. According to Table 5, the relationship between SNC and indicator *a*9 (social relationships with others) should be removed from the initial model as its loading is very small (0.19). Meanwhile, the relationship is estimated as not significant as shown in Table 5, which reflects that the group and team communication would be more important than individual communication [134]. Furthermore, frequent occupational health and safety education training could strengthen health self-protection [135]. Therefore, the relationship between *a*11 and *a*1 should be added. As a result, the improved model is proposed as shown in Figure 4. The estimates of pathway coefficients are presented in Figure 4. According to the estimation, the improved model shows a very good model fit (χ^2^/D*f* = 1.130, CFI = 0.996, NFI = 0.967, RMSEA = 0.033). The improved model has a high GOF measure. In fact, the CFI and RMSEA are the most important and reported indices to indicate the model fitness [136]. For the improved model, the value of CFI and RMSEA indicates that the model fit can meet the requirements of further analysis according to Doloi and other experts [137]. The results of the model evaluation are shown in Table 6. All proposed relationships are estimated as significant. Therefore, all measured values are mainly acceptable and meet the requirements. The model is feasible, and there is no necessity to adjust the model to improve the level of GOF measure.

The latent variables describing the P&M health can be measured by *a*1–*a*4 and *a*7 in an improved model. All indicators are contributing greatly to the P&M health, but in different levels. The most significant impact is from *a*4 (frequency of physical examination), which indicates that timely health assessment is critical for enhancing the health status of construction workers [110]. Moreover, rest time (*a*3) also receives a high factor loading in this package. Enough rest time is recognized as a necessary method to improve labour health, efficiency and productivity [23]. Meanwhile, the impacts of self-protection consciousness, daily working hours and the occurrence frequency of anxious and upset mood on the P&M health cannot be neglected because their relationships are statistically significant, as shown in Table 6.

The latent variables describing the social capital and network can be measured by *a*8, *a*10 and *a*11. Indicators in this package all have important different contributions to the social capital and network. The frequency of check by the government (*a*8) receives the highest factor loading in the improved model, which indicates that support from the government should be the most important to strengthen the social status of construction workers and their efficiency and productivity [115]. At the same time, the supports from construction companies including protection measures (*a*10) and health training (*a*11) are also similarly significant.

The latent variables describing the work efficiency and productivity can be measured by *a*12, *a*13 and *a*14. Indicators in this package all have strong positive impacts on the work efficiency and productivity. Quality of finished work (*a*12) was the most important measure of work efficiency and productivity according to Figure 4 and Table 6. Work progress and work attitude also can greatly influence the work efficiency and productivity. Especially for work attitude, the positive relationship between them can be used to educate and train the construction workers to improve work performance [121].

## 6. Discussion and Research Findings

### 6.1. Key Relationships among Health, SNC and Work Efficiency and Productivity

The structural components of the initial model are presented in Figure 4. The relationships among three variables are found to be significant in the initial model as shown in Figure 4 and Table 6. As presented before, there are three hypothetical relationships among three variables.

The first hypothesis (H1) is that P&M health has a positive influence on work efficiency and productivity. H1 is supported by the results of SEM (P&M health “work efficiency and productivity, 0.67), which means the improvement of work performance should depend on the enhancement of P&M health for construction workers. Moreover, good health is essential to the success of a construction project. Protecting construction workers from ill health is an important sign of a project that is likely to grow and thrive.

The second hypothesis (H2) is that the strong social supports from social capital and the network have a positive influence on construction work efficiency and productivity. H2 is also supported by the results of SEM (social capital and network work efficiency and productivity, 0.25), which means management of construction projects should provide more supports to construction workers from the perspective of government and enterprises. Due to the vulnerable features of the construction workers with temporary work on site and fragile relationship with employers, more frequent government supervision, more effective work protection and more frequent health training are needed.

The third hypothesis (H3) is that the social capital and network have a positive influence on P&M health for construction workers. H3 is also strongly supported by the results of SEM (social capital and network P&M health, 0.91), which means social capital and the network can influence health directly by activating cognitive systems and indirectly by giving a sense of coherence and meaningfulness. Actually, construction workers with access to cognitive and structural social capital have a higher odds ratio for good health compared to workers with no access to these forms of social capital. Therefore, individual’s aspirations should be realized and the social needs should be satisfied to change or cope with the environment to facilitate health promotion, which is the process of enabling people to increase control over, and to improve, their health [138]. Furthermore, the impact of occupational health training on self-protection is tested to be significantly positive, which also supports that social capital and network have a positive influence on P&M health.

### 6.2. The Way to Improve the Construction Work Efficiency and Productivity

In addition, comparing factor loadings among these three relationships, the most important and direct method to improve the construction work efficiency and productivity is to promote P&M health. Compared to direct impacts of social capital and network on the work efficiency and productivity, the direct impacts of P&M health on the work efficiency and productivity are larger. Frequent physical examination and a reasonable rest schedule and time are the best way to improve the health and work efficiency and productivity.

Meanwhile, strengthening self-protection consciousness, keeping reasonable working hours and reducing the frequency of anxious and upset mood also comprise an effective way to improve the health and work efficiency and productivity for construction workers.

Considering the direct impacts of social capital and network on the work efficiency and productivity, increasing the social supports for the construction workers could also improve work efficiency and productivity. Improving the frequency of checks by the government can enhance the supervision and support of government and facilitate a healthy working environment. Enhancing the protection measures and occupational health training provided by companies are other ways of improving work efficiency and productivity from the perspective of direct impacts of social capital and network.

Moreover, the social capital and network can indirectly affect the work efficiency and productivity by influencing P&M health. Social support from the social capital and network of construction workers has been linked to many benefits for P&M health. Social support can be associated with increased psychological well-being in the construction workplace to reduce psychological distress (e.g., anxious and upset mood). Social support also has been found to promote self-protection consciousness with psychological adjustment to regulate emotional responses that arise from a stressful event at a construction site. Furthermore, social support from the enterprise can provide frequent physical examination and has a clearly demonstrated link to physical health outcomes in individuals, which can help workers learn their health information and enhance self-protection consciousness. All impacts from social capital and network will be transferred by P&M health to improve the work efficiency and productivity.

### 6.3. Suggestions for Government, Project Managers and Construction Workers

As mentioned above, the main important findings in this study are the identification of the key relationships among construction workers’ P&M health, SNC and work efficiency and productivity. Many useful suggestions can be drawn from the research findings.

Governments should put more efforts into improving the legal and policy environment to protect construction workers’ health physically and mentally, as well as provide effective governmental supports for construction workers, which would finally improve the construction work efficiency and productivity. The regulation of the occupational health for construction workers should be strengthened including improving the frequency of health checks by the government and requiring the construction companies to control the construction workers’ daily working hours, enhancing the health protection and training.

For construction companies, many detailed measures should be conducted during the project management. Providing enough protection measures should be more important, which can include health insurance, regular physical examination, health subsidies for high temperature and cold weather, necessary protective equipment and fixed vacations for construction workers. Addressing the workers should be another significant way to improve the health and construction work efficiency and productivity including health training, daily working hours and rest time.

For construction workers, learning more information about health and enhancing their social relationships would be helpful with their work efficiency and productivity. Self-protection consciousness should be firstly concerned with the support of government and construction companies. Strengthening the self-control of rest time and emotions should be very important. Moreover, effective emotional self-control would reduce daily life disorders, social communication complexity and goal-directed blindness. Meanwhile, conscientiously participating in health training should be given more attention by construction workers, which can help them learn more knowledge and information about their health.

## 7. Conclusions

This paper investigated the indicators of construction workers’ health, SNC and work efficiency and productivity through a literature review. The data on these variables and different indicators were collected from construction sites in Nanjing, China. A conceptual model was proposed based on the prior studies to explain the theoretical relationships among construction workers’ health, SNC and work efficiency and productivity. An SEM was conducted to identify the major factors affecting work efficiency and productivity, to test the proposed conceptual model, as well as the relationships in the model.

A reliability analysis was conducted to test the internal consistency of the variables by confirming that opinions of different construction workers on variables and different indicators are consistent. The survey results demonstrated that rest time and frequency of physical examination are more important to improve construction workers’ health compared to self-protection consciousness, daily working hours and occurrence frequency of anxious and upset mood. For construction social capital and network, government check, protection measures and health training are almost of the same importance for enhancing social supports. Moreover, construction work quality, progress and attitude can measure the work efficiency and productivity well, in which quality should be the most important.

The SEM method was also used to test whether the hypothesized model correlated with data collected from the survey. The results of the SEM on the improved model reflect a strong relationship, which indicates that all identified indicators can measure their corresponding variables well. The three hypothetical relationships among factor packages were tested by the SEM results. H1 (P&M health has a positive influence on work efficiency and productivity), H2 (the SNC has a positive influence on work efficiency and productivity) and H3 (SNC has a positive influence on P&M health) were positively supported by the SEM method. The results of performing SEM indicated that the direct impacts of construction workers’ P&M health on work efficiency and productivity were identified to be much more important than that of the SNC. In addition, construction workers’ social capital can indirectly influence the work efficiency and productivity by affecting the construction workers’ P&M health. Therefore, three ways to improve construction workers’ work efficiency and productivity were proposed by the study. Furthermore, many useful suggestions can be drawn from the research findings from the perspective of government, construction companies and workers.

The identified indicators and relationship would be very useful for construction work efficiency and productivity assessment, health management and safety management from the perspective of the construction workers. Although this study obtained very useful findings regarding construction workers’ health, SNC and work efficiency and productivity, more data and information should be obtained from construction sites to avoid some deviations; also because of the limited condition, the workers were not stratified based on work ability index; moreover, the suggestion has been raised based on the factors, their interaction regularity is not analysed, this may require the simulation to go further. Meanwhile, the relationships between construction workers’ health and SNC should further be considered with the impacts of workers’ behaviour, which may also influence work efficiency and productivity. Their relationships should be clarified by further research.

## Figures and Tables

**Table 1:** Corresponding model variables. P&M, physical and metal (health); SNC, social network and capital.

Latent Variables	No.	Observed Variables	References
P&M health	*a*1	Self-protection consciousness	[107]
	*a*2	Daily working hours	[108]
	*a*3	Rest time	[109]
	*a*4	Frequency of physical examination	[110]
	*a*5	Sleeping time	[111]
	*a*6	Working years	[112]
	*a*7	Occurrence frequency of anxious and upset mood	[113,114]
SNC	*a*8	Frequency of check by government	[115]
	*a*9	Social relationships with others	[117]
	*a*10	Protection measures provided by companies	[118]
	*a*11	Specific occupational health training	[119]
Work efficiency and productivity	*a*12	Quality of finished work	[120]
	*a*13	Work progress	[120]
	*a*14	Work attitude	[121]

**Table 2:** The content of the questionnaire.

Latent Variables	No.	Observed Variables	Questions
P&M health	*a*1	Self-protection consciousness	Do you usually consciously do some health self-protection measures?
			1- *Unconscious*; 2- *Slight attention*; 3- *Conscious*; 4- *Emphasis*; 5- *Very concerned*
	*a*2	Daily working hours	How long do you work every day?
			1- *11 hours or more*; 2- *10~11 hours*; 3- *8~9 hours*; 4- *10~11 hours*; 5- *8 hours or less*
	*a*3	Rest time	How much time construction workers spend on entertainment every day?
			1- *Half hour or less*; 2- *0.5~1 hours*; 3- *1~1.5 hours*; 4- 1.5*~2 hours*; 5- *2 hours or more*
	*a*4	Frequency of physical examination	What is your physical examination frequency?
			1- *Never*; 2- *Once every three years or more*; 3- *Once every two years*; 4- *Once a years*; 5- *Once every six months*
	*a*5	Sleeping time	How about your sleep status on construction site?
			1- *Often insomnia*; 2- *Occasional insomnia*; 3- *Easy to fall asleep, but difficult to sleep*; 4- *Easy to sleep, but feels lack of sleep*; 5- *Sufficient sleep*
	*a*6	Working years	How many years do you have been working for?
			1- *3 years or less*; 2- *3~5 years*; 3- *5~10 years*; 4- *10~15 years*; 5- *15 years or more*
	*a*7	Occurrence frequency of anxious and upset mood	How often do you become impatient, depressed and worried?
			1- *Never*; 2- *Occasionally*; 3- *Interruptedly*; 4- *Often*; 5- *Continued to appear*
SNC	*a*8	Frequency of check by government	What is the inspection frequency by the relevant government departments?
			1- *Never*; 2- *Once every three years or more*; 3- *Once every two years*; 4- *Once a years*; 5- *Once every six months*
	*a*9	Social relationships with others	Do you encounter any of the following situations in your social communication?
			A. Feels that most people are not trustworthy B. Feels uncomfortable when he gets along with the opposite sex C. Feels that people don’t understand you and don’t feel friendly to you D. Feels uncomfortable when in movie theatre or shopping malls E. Worried about his clothes and his posture
			1- *Select one option*; 2- *Select two options*; 3- *Select three options*; 4- *Select four options*; 5- *Select five options*
	*a*10	Protection measures provided by companies	Which of the following health protection measures has been provided to you by the companies where you work?
			A. Health insurance B. Regular physical examination C. Health subsidies for high temperature and cold weather D. Necessary protective equipment E. Fixed vacation
			1- *Select one option*; 2- *Select two options*; 3- *Select three options*; 4- *Select four options*; 5- *Select five options*
	*a*11	Specific occupational health training	Frequency of occupational health and safety education training provided by companies
			1- *Never*; 2- *At least once a year*; 3- *At least twice a year*; 4- *At least three times a year*; 5- *At least four times a year*
Work efficiency and productivity	*a*12	Quality of finished work	What is the average degree of rework for the part that you are responsible for?
			1- *Extensive rework*; 2- *Extensive repair*; 3- *Local repair*; 4- *Small scale repair*; 5- *Basically no rework*
	*a*13	Work progress	What is the percentage of finished tasks compared with the planned tasks?
			1- *60% or less*; 2- *70%*; 3- *80%*; 4- *90%*; 5- *100%*
	*a*14	Work attitude	What about your attitudes towards daily work?
			1- *Don’t care*; 2- *Cannot complete the tasks on time*; 3- *Barely finished his work*; 4- *Completes the work seriously*; 5- *Completes the task, looking for more efficient methods*

**Table 3:** Project total statistical scale.

Factors	*a*1	*a*2	*a*3	*a*4	*a*5	*a*6	*a*7
Cronbach’s Alpha if Item Deleted	0.894	0.908	0.902	0.895	0.909	0.919	0.905
Factors	*a*8	*a*9	*a*10	*a*11	*a*12	*a*13	*a*14
Cronbach’s Alpha if Item Deleted	0.898	0.908	0.899	0.897	0.901	0.902	0.896

**Table 4:** The recommended level of goodness-fit (GOF) measures. CFI, comparative fit index; NFI, normal fit index.

Goodness of Fit Measure	Recommended Level of GOF Measure
χ^2^/D*f*	From 1–5
CFI	0 (no fit) to 1 (perfect fit)
NFI	0 (no fit) to 1 (perfect fit)
(RMSEA)	<0.05 indicate very good fit (threshold level = 0.1)

**Table 5:** Coefficient estimation of parameters in the initial model. CR, critical ratio.

Factors and Indicators	Relationships	Factors and Indicators	Estimate	Standard Error	CR	*p*	Standardized Estimate
Work efficiency and productivity	←	P&M health	0.365	0.084	4.334	***	0.583
Work efficiency and productivity	←	SNC	0.293	0.103	2.833	***	0.355
P&M health	←	SNC	1.243	0.101	12.307	***	0.944
*a*1	←	P&M health	1.000				0.884
*a*2	←	P&M health	0.392	0.066	5.971	***	0.500
*a*3	←	P&M health	0.895	0.090	9.995	***	0.727
*a*4	←	P&M health	1.159	0.085	13.614	***	0.860
*a*5	←	P&M health	0.297	0.088	3.392	***	0.303
*a*7	←	P&M health	0.475	0.074	6.438	***	0.531
*a*8	←	SNC	1.088	0.115	9.653	***	0.759
*a*9	←	SNC	0.187	0.086	2.174	0.030	0.189
*a*10	←	SNC	0.839	0.084	10.045	***	0.783
*a*11	←	SNC	1.000				0.784
*a*12	←	Work efficiency and productivity	1.000				0.803
*a*13	←	Work efficiency and productivity	0.988	0.117	8.419	***	0.721
*a*14	←	Work efficiency and productivity	1.214	0.119	10.205	***	0.836

While the absolute value of CR should be more than 2.58, the parameters are estimated at the 0.01 significance level, with *p* shown with “***”.

**Table 6:** Coefficient estimation of parameters in the improved model.

Factors and Indicators	Relationships	Factors and Indicators	Estimate	Standard Error	CR	*p*	Standardized Estimate
Work efficiency and productivity	←	P&M health	0.660	0.208	3.173	***	0.672
Work efficiency and productivity	←	SNC	0.233	0.054	4.315	***	0.254
P&M health	←	SNC	0.987	0.137	7.205	***	0.912
*a*1	←	P&M health	1.000				0.585
*a*2	←	P&M health	0.574	0.120	4.786	***	0.484
*a*3	←	P&M health	1.385	0.198	6.979	***	0.743
*a*4	←	P&M health	1.823	0.237	7.692	***	0.895
*a*7	←	P&M health	0.723	0.139	5.201	***	0.534
*a*8	←	SNC	1.149	0.114	7.956	***	0.744
*a*10	←	SNC	0.932	0.107	8.743	***	0.809
*a*11	←	SNC	1.000				0.730
*a*12	←	Work efficiency and productivity	1.000				0.832
*a*13	←	Work efficiency and productivity	0.973	0.110	8.882	***	0.742
*a*14	←	Work efficiency and productivity	1.097	0.114	9.597	***	0.786
*a*1	←	*a*11	0.450	0.084	5.368	***	0.384
